# Association between tensin 1 and p130Cas at focal adhesions links actin inward flux to cell migration

**DOI:** 10.1242/bio.016428

**Published:** 2016-03-30

**Authors:** Zhihai Zhao, Song Hui Tan, Hiroaki Machiyama, Keiko Kawauchi, Keigo Araki, Hiroaki Hirata, Yasuhiro Sawada

**Affiliations:** 1 Mechanobiology Institute, National University of Singapore, 5A Engineering Drive 1, 117411, Singapore; 2 Department of Biological Sciences, National University of Singapore, 14 Science Drive 4, 117543, Singapore; 3 Department of Biomedical Engineering, National University of Singapore, 9 Engineering Drive 1, Block EA, #03-12, 117575, Singapore; 4 Department of Rehabilitation for the Movement Functions, National Rehabilitation Center for Persons with Disabilities, 4-1 Namiki, Tokorozawa, Saitama 359-8555, Japan; 5 Laboratory for Mechanical Medicine, Locomotive Syndrome Research Institute, Nadogaya Hospital, 687-4 Nadogaya, Kashiwa, Chiba 277-0032, Japan

**Keywords:** Tensin 1, P130Cas, Actin cytoskeleton, Cell migration, Focal adhesion

## Abstract

Cell migration is a highly dynamic process that plays pivotal roles in both physiological and pathological processes. We have previously reported that p130Cas supports cell migration through the binding to Src as well as phosphorylation-dependent association with actin retrograde flow at focal adhesions. However, it remains elusive how phosphorylated Cas interacts with actin cytoskeletons. We observe that the actin-binding protein, tensin 1, co-localizes with Cas, but not with its phosphorylation-defective mutant, at focal adhesions in leading regions of migrating cells. While a truncation mutant of tensin 1 that lacks the phosphotyrosine-binding PTB and SH2 domains (tensin 1-SH2PTB) poorly co-localizes or co-immunoprecitates with Cas, bacterially expressed recombinant tensin 1-SH2PTB protein binds to Cas *in vitro* in a Cas phosphorylation-dependent manner. Furthermore, exogenous expression of tensin 1-SH2PTB, which is devoid of the actin-interacting motifs, interferes with the Cas-driven cell migration, slows down the inward flux of Cas molecules, and impedes the displacement of Cas molecules from focal adhesions. Taken together, our results show that tensin 1 links inwardly moving actin cytoskeletons to phosphorylated Cas at focal adhesions, thereby driving cell migration.

## Abstract (precis)

**Summary:** Tensin 1 links phosphorylated Cas to actin inward flux, facilitating the force transmission from the motile system (i.e. actomyosin contraction) to the stationary parts (i.e. adhesion complexes) in migrating cells.

## INTRODUCTION

Cell migration is a complex biological process that supports or underlies a diverse range of physiological or pathological events and functions including embryonic development, immune responses and tumor metastasis. While the continuous inward movement of actin filaments provides driving forces for cell migration, a transmission mechanism is required between the motile and stationary parts in the cytoskeleton-adhesion linkage (Giannone et al., 2009; Gardel et al., 2010). We have recently reported that p130Cas (Crk-associated substrate, hereafter referred to as Cas) is involved in such a transmission as it supports cell migration by linking the actomyosin-driven actin retrograde flux to the cell-extracellular matrix adhesions (Machiyama et al., 2014). We have shown that both the Src-mediated tethering and the actomyosin-driven displacement of Cas are involved in the mechanism that drives cell migration. Detailed analysis regarding the process of Cas displacement has revealed that upon phosphorylation of the substrate domain (CasSD), Cas molecules move inward in association with the actin retrograde flux, and get dissociated from focal adhesions. However, it remains unclear how actin cytoskeletons are linked to Cas molecules with phosphorylated CasSD.

In search for such a molecule or molecular complex that can interact with both actin filaments and phosphorylated Cas simultaneously, we consolidated previous reports on actin-associating proteins that may bind to phosphotyrosine-containing polypeptides. Tensin 1 is a focal adhesion protein that has been reported to associate with actin filaments through its actin-binding domains located in the amino-terminal and central regions of the molecule (Davis et al., 1991; Auger et al., 1996; Wavreille and Pei, 2007). In addition, tensin 1 has the phosphotyrosine-binding SH2 (Src homology 2) and PTB (phosphotyrosine-binding) domains (Schlessinger and Lemmon, 2003). It has been documented that tensin 1 co-immunoprecipitates with Cas *in vitro*, although physiological role of the tensin 1-Cas interaction has not been defined (Hall et al., 2010). Therefore, we tested tensin 1 as a molecule that links actin cytoskeletons to phosphorylated Cas.

## RESULTS AND DISCUSSION

### Cas, but not its phosphorylation-defective mutant, co-localizes with tensin 1 at focal adhesions in the regions close to leading edge

**Fig. 1.**
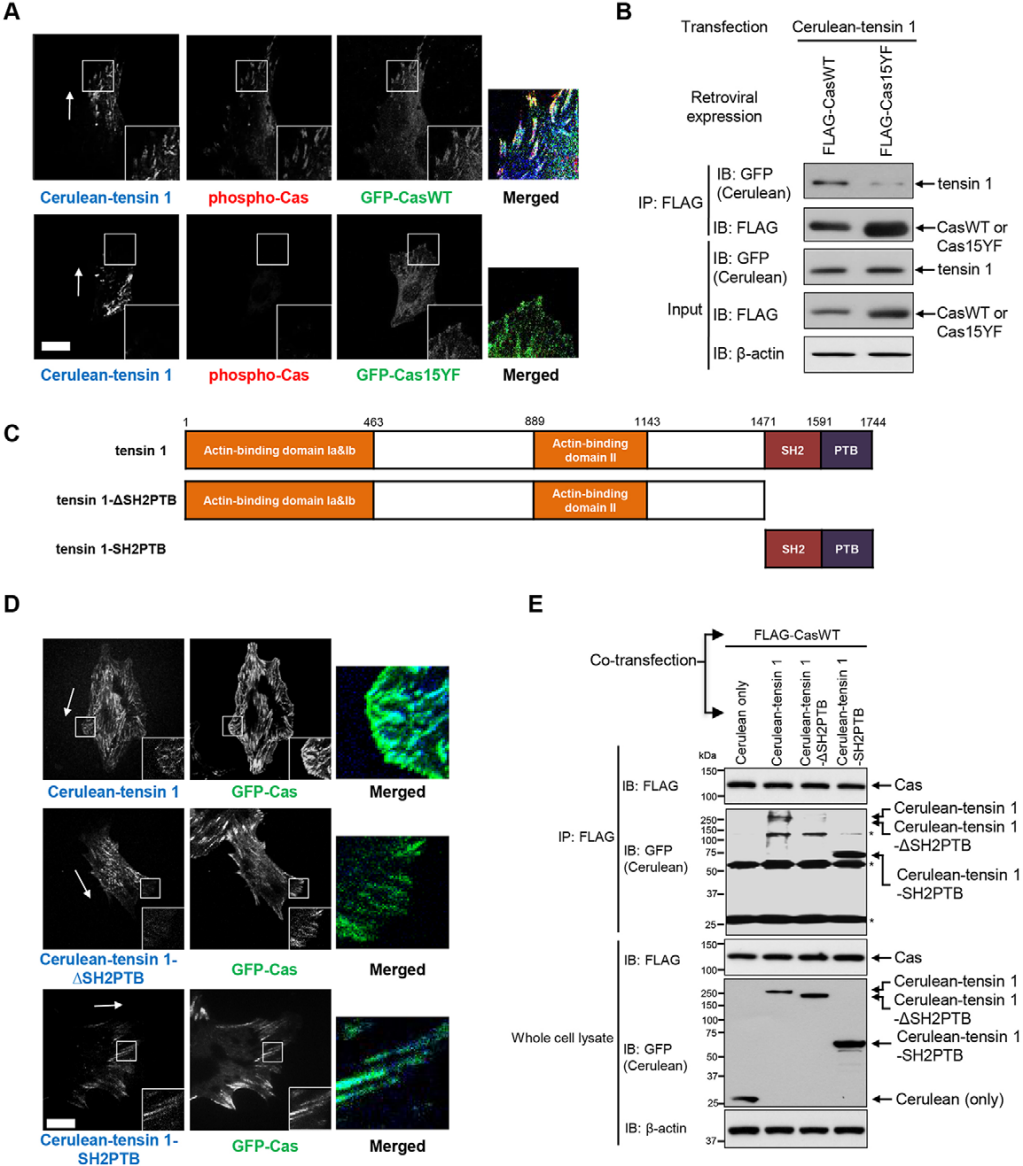
**Cas co-localizes with tensin 1 at focal adhesions in the regions close to leading edge of migrating cells depending on Cas phosphorylation.** (A) Confocal images of CasKO MEFs expressing Cerulean-tensin 1 together with either GFP-CasWT (top panels) or GFP-Cas15YF (bottom panels) after immunostaining against phosphorylated Cas. Monolayers of GFP-CasWT- or GFP-Cas15YF-expressing CasKO MEFs transfected with Cerulean-tensin 1 were subjected to wound healing experiments. Migrating cells were fixed, subjected to immunofluorescence staining and viewed with a confocal microscope. Magnified images of boxed regions are shown in insets. Merged images of the boxed regions are also shown (blue: Cerulean-tensin 1, red: phospho-Cas, green: GFP-CasWT or GFP-Cas15YF). Scale bar: 5 μm. Arrows indicate the direction of cell migration. (B) Anti-FLAG immunoprecipitates from the lysate of HEK293T cells expressing Cerulean-tensin 1 together with either FLAG-CasWT or FLAG-Cas15YF were analyzed by immunoblotting. HEK293T cells were co-transfected with Cerulean-tensin 1 together with either FLAG-CasWT or FLAG-Cas15YF. 24 h after transfection, cells were lysed and subjected to anti-FLAG immunoprecipitation followed by SDS-PAGE and anti-FLAG or anti-GFP immunoblotting. Anti-β-actin blot provided a loading control. Cerulean-tagged proteins were detected using an anti-GFP antibody that cross-reacted to Cerulean (anti-GFP blot). (C) Schematic representation of truncated mutants of tensin 1. SH2, Src-homology 2 domain; PTB, phosphotyrosine-binding domain. (D) Confocal images of CasKO MEFs expressing GFP-CasWT together with Cerulean-tensin 1 (top panels), Cerulean-tensin 1-ΔSH2PTB (middle panels) or Cerulean-tensin 1-SH2PTB (bottom panels). Monolayers of GFP-Cas-expressing CasKO MEFs transfected with either Cerulean-tensin 1, Cerulean-tensin 1-ΔSH2PTB or Cerulean-tensin 1-SH2PTB were subjected to wound healing experiments. Migrating cells were then viewed with a confocal microscope. Magnified images of boxed regions are shown in insets. Merged images of the boxed regions are also shown (blue: Cerulean-tensin 1, Cerulean-tensin 1-ΔSH2PTB or Cerulean-tensin 1-SH2PTB, green: GFP-CasWT). Scale bar: 5 μm. Arrows indicate the direction of cell migration. (E) Anti-FLAG immunoprecipitates from the lysate of HEK293T cells expressing FLAG-CasWT together with either Cerulean, Cerulean-tensin 1, Cerulean-tensin 1-ΔSH2PTB or Cerulean-tensin 1-SH2PTB were analyzed by immunoblotting. HEK293T cells were co-transfected with FLAG-CasWT together with either Cerulean only, Cerulean-tensin 1, Cerulean-tensin 1-ΔSH2PTB or Cerulean-tensin 1-SH2PTB. 24 h after transfection, cells were lysed and subjected to anti-FLAG immunoprecipitation followed by SDS-PAGE and anti-FLAG or anti-GFP immunoblotting. Anti-β-actin blot provided a loading control. Cerulean-tagged proteins were detected with anti-GFP blot as in B. Asterisks indicate the IgG bands (∼26 kD and ∼52 kD) and unidentified non-specific bands (∼125 kD). B and E represent one of three independent experiments with similar results.

We first examined whether tensin 1 co-localized with Cas in relation to Cas phosphorylation. To this end, we analyzed mouse embryonic fibroblasts (MEFs) derived from Cas knockout (CasKO) mice (Honda et al., 1998) expressing Cerulean-tagged tensin 1 (Cerulean-tensin 1) together with green fluorescent protein (GFP)-tagged Cas wild-type (GFP-CasWT) or its phosphorylation-defective mutant (GFP-Cas15YF) (Machiyama et al., 2014) using a confocal microscope. We observed that GFP-CasWT and Cerulean-tensin 1 co-localized at focal adhesions in the regions close to leading edges of migrating cells (Fig. 1A, top panels). In contrast, Cerulean-tensin 1 was poorly localized at focal adhesions in GFP-Cas15YF-expressing CasKO MEFs, and there was no apparent co-localization of Cerulean-tensin 1 and GFP-Cas15YF (Fig. 1A, bottom panels). Furthermore, indirect immunofluorescence staining using an anti-phospho-Cas antibody (pCas-165) (Fonseca et al., 2004; Sawada et al., 2006) showed a correlation between GFP-CasWT-Cerulean-tensin 1 co-localization and Cas phosphorylation (Fig. 1A, top panels). These findings suggest that tensin 1 co-localizes with Cas at focal adhesions in the leading regions of migrating cells, depending on Cas phosphorylation.

To biochemically analyze the interaction between tensin 1 and Cas, we conducted a co-immunoprecipitation assay using human embryonic kidney (HEK) 293T cells expressing Cerulean-tensin 1 together with FLAG-tagged wild-type Cas (FLAG-CasWT) or Cas15YF (FLAG-Cas15YF). We found that Cerulean-tensin 1 was co-immunoprecipitated with FLAG-CasWT (Fig. 1B, left lane). In contrast, Cerulean-tensin 1 was poorly co-immunoprecipitated with FLAG-Cas15YF (Fig. 1B, right lane). These results suggest that the interaction between Cas and tensin 1 is dependent on Cas phosphorylation, while the apparently faint association between FLAG-Cas15YF and Cerulean-tensin 1 leaves the possibility of the existence of an additional mechanism of Cas-tensin 1 binding independent of Cas phosphorylation.

### The SH2PTB domains of tensin 1 are responsible for its interaction with phosphorylated Cas

The SH2 and PTB domains of tensin 1 (hereafter referred to as tensin 1-SH2PTB) are reportedly responsible for its association with phosphotyrosine-containing proteins (Davis et al., 1991; Auger et al., 1996; Wavreille and Pei, 2007). We therefore examined whether tensin 1-SH2PTB played a significant role in the association between phosphorylated Cas and tensin 1 by testing tensin 1 (full-length) and its deletion mutants, tensin 1-ΔSH2PTB and tensin 1-SH2PTB (Fig. 1C). In contrast to Cerulean-tensin 1 (full-length) (Fig. 1D, top panels) and Cerulean-tensin 1-SH2PTB (Fig. 1D, bottom panels), Cerulean-tensin 1-ΔSH2PTB was poorly localized at focal adhesions and did not exhibit apparent co-localization with GFP-CasWT (Fig. 1D, middle panels) in the leading regions of migrating cells when they were co-expressed in CasKO MEFs. Consistently, co-expression experiments using HEK293T cells revealed that Cas co-immunoprecipitated with tensin 1 and tensin 1-SH2PTB (Fig. 1E, lanes 2 and 4), but very poorly with tensin 1-ΔSH2PTB (Fig. 1E, lane 3). These results indicate that SH2PTB domains are crucial for tensin 1 to associate with Cas at focal adhesions in the leading regions, while the apparent co-localization of GFP-CasWT and Cerulean-tensin 1 or Cerulean-tensin 1-ΔSH2PTB in the central regions of cells (Fig. 1D, top and middle panels) may denote Cas-tensin 1 interaction independent of the SH2PTB domains of tensin 1. Based on the significance of Cas dynamics at focal adhesions in cell migration (Machiyama et al., 2014), we specifically focus, in this study, on behaviors of Cas and tensin 1 in the leading regions.

We then examined whether tensin 1-SH2PTB alone bound to Cas in a CasSD phosphorylation-dependent manner. We prepared a bacterially expressed recombinant protein in which tensin 1-SH2PTB was fused to GST (glutathione-S transferase), and conducted a GST pull-down assay. We found that FLAG-Cas was brought down with GST-tensin 1-SH2PTB (Fig. S1, lane 2). Furthermore, FLAG-Cas ‘pull-down’ with GST-tensin 1-SH2PTB was remarkably enhanced when Cas phosphorylation was increased by treating the cells with phenylarsine oxide (PAO), the tyrosine phosphatase inhibitor (Fig. S1, lane 4). These findings support the notion that tensin 1 associates with Cas through the binding between tensin 1-SH2PTB and phosphorylated Cas.

### Exogenous expression of tensin 1-SH2PTB interferes with Cas-driven cell migration

**Fig. 2.**
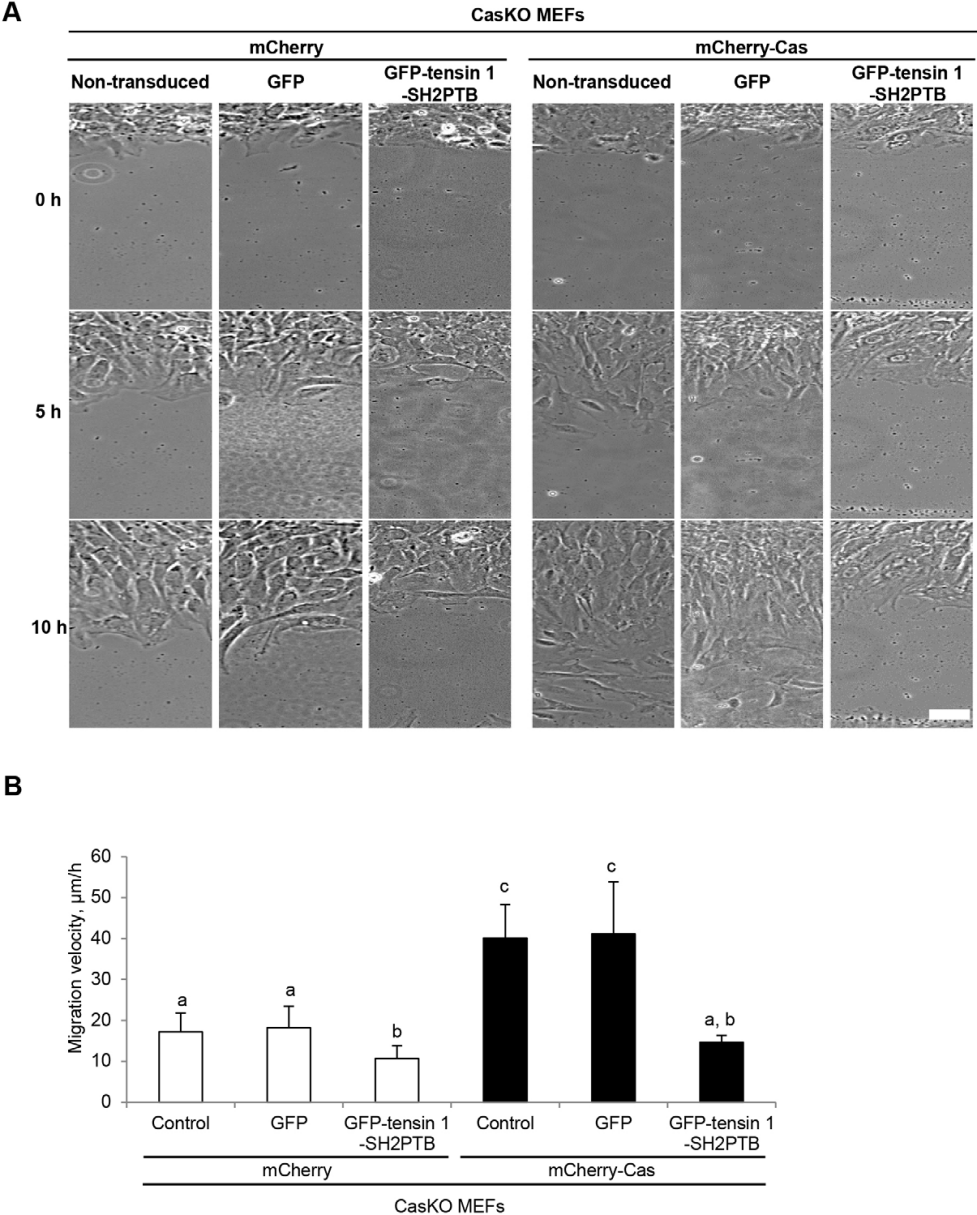
**Exogenous expression of tensin 1-SH2PTB interferes with Cas-driven cell migration.** (A) Representative images of cells either non-transduced, transduced with GFP or GFP-tensin 1-SH2PTB at 0, 5 and 10 h after wounding. Scale bar: 50 μm. Monolayers of mCherry-(left panels) or mCherry-Cas-(right panels) expressing CasKO MEFs, either non-transduced or adenovirally transduced with GFP only or GFP-tensin 1-SH2PTB, were subjected to wound healing experiments. Migrating cells were viewed with a microscope. (B) The cell migration velocity was defined by the distance over which nuclei moved during recording. Error bars represent s.e.m. The attached letters (a,b,c) indicate the groups statistically determined by a one-way ANOVA followed by Tukey–Kramer post hoc testing (*n*>30 in each group). Columns that do not share the same group letters are significantly different from each other (*P*<0.01), whereas there is no significant difference between the letter-sharing columns. Similar results were obtained from three independent experiments.

Tensin 1 has been reported to positively regulate cell migration (Chen et al., 2002; Chen and Lo, 2003). Consistently, we observed that the silencing of tensin 1 impeded cell migration in a wound healing assay (Fig. S2). We postulated that the tensin 1-SH2PTB-mediated interaction between tensin 1 and Cas (Fig. 1E,F) might be responsible for the linkage of phosphorylated Cas to actin cytoskeletons that drives cell motility (Machiyama et al., 2014). To test this, we examined whether the exogenous expression of tensin 1-SH2PTB protein (Fig. S3) that binds to phosphorylated Cas (Fig. S1) but lacks the actin-binding motifs modulated Cas-driven cell migration. As shown in Fig. 2A and B, the expression of mCherry-Cas in CasKO MEFs significantly promoted the cell migration (compare columns 1 and 4 in Fig. 2B). While the expression of GFP-tensin 1-SH2PTB significantly slowed down the migration of CasKO MEFs expressing either mCherry alone (columns 1, 2 and 3 in Fig. 2B) or mCherry-Cas (columns 4, 5 and 6 in Fig. 2B), it was more prominent in the latter (CasKO MEFs expressing mCherry-Cas). The negative effect of GFP-tensin 1-SH2PTB expression on the migration of CasKO MEFs indicates that the SH2PTB domains of tensin 1 is also involved in Cas-independent regulation of cell motility. Nevertheless, the promoting effect of mCherry-Cas expression on cell migration was eliminated by the expression of GFP-tensin 1-SH2PTB (compare columns 3 and 6 in Fig. 2B). This suggests that tensin 1 serves as a major mediator of the association between phosphorylated Cas and actin cytoskeletons during cell migration.

### Exogenous expression of tensin 1-SH2PTB slows down the inward flux of Cas molecules, but not the retrograde actin flow

**Fig. 3.**
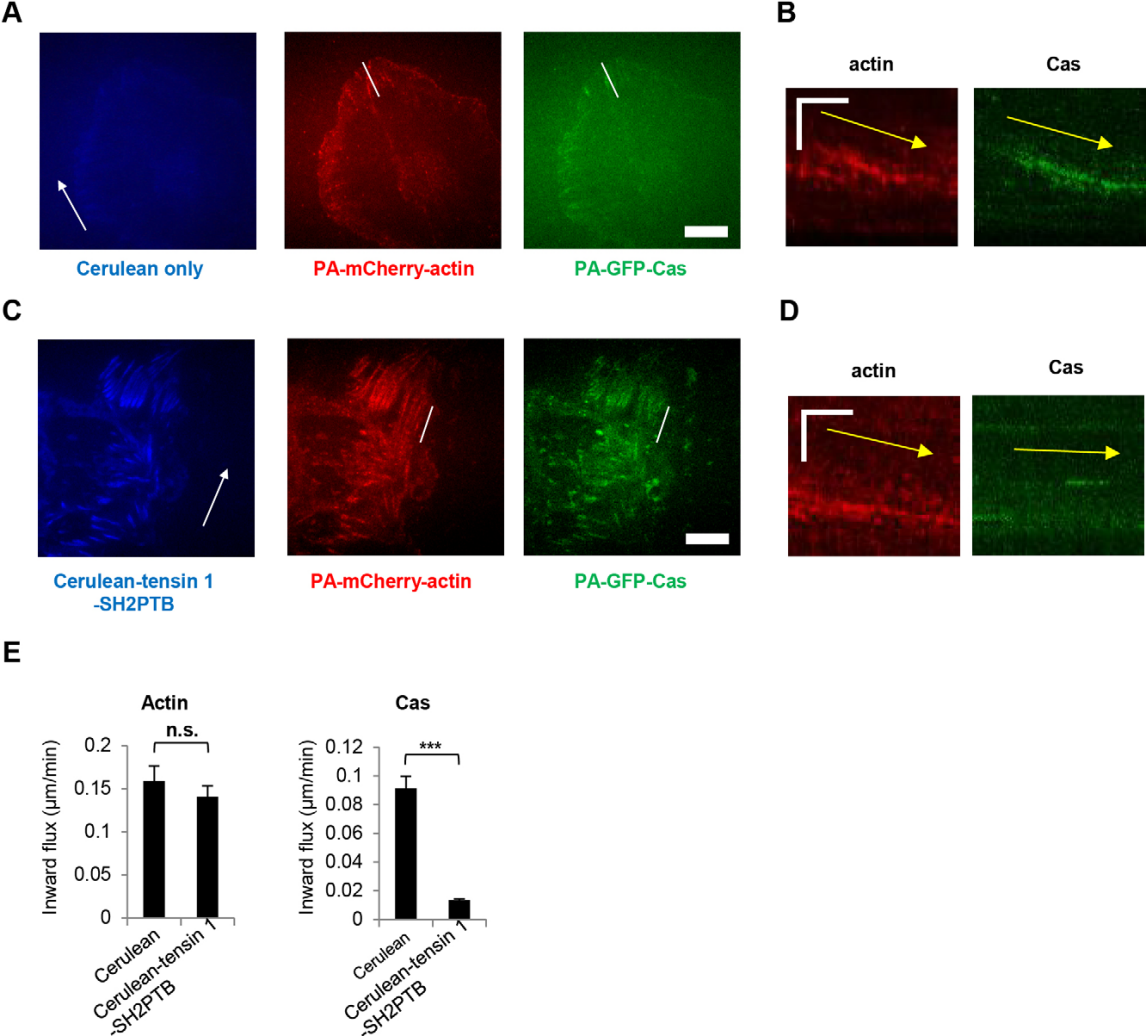
**Exogenous expression of tensin 1-SH2PTB disrupts the correlation between the inward fluxes of actin and Cas molecules.** (A,C) Representative images of migrating cells expressing PA-GFP-Cas and PA-mCherry-actin together with either Cerulean only (A) or Cerulean-tensin 1-SH2PTB. (C). Monolayers of PA-GFP-Cas- and PA-mCherry-actin-expressing CasKO MEFs transfected with either Cerulean only or Cerulean-tensin 1-SH2PTB were subjected to wound healing experiments. Migrating cells were analyzed by PA-TIR-FSM as described in Materials and Methods. Scale bar: 5 μm. Arrows indicate the direction of cell migration. (B,D) Kymographs drawn on the movement of actin and Cas molecules along the white dashed lines in A and C. The arrows represent the retrograde movement of actin and Cas molecules. Vertical scale: 5 min; horizontal scale: 2 μm. (E) The rates of PA-GFP-Cas and PA-mCherry-actin movement were quantified based on the kymographs in B and D. Data show the mean±s.e.m.;***P<0.001; n.s.: not significant (*n*>20 in each group); Student's *t*-test. Similar results were obtained from three independent experiments.

We previously reported that the inward movement of Cas molecules correlated with the myosin-driven actin retrograde flow that promoted focal adhesion disassembly and cell migration (Machiyama et al., 2014). We therefore examined whether the exogenous expression of tensin 1-SH2PTB, which impeded Cas-driven cell migration (Fig. 2B), disrupted the correlation between the actin retrograde flow and the movement of Cas molecules. The movement of Cas molecules and the retrograde flow of actin were monitored at focal adhesions in the leading regions by tracking speckles of photoactivatable GFP-Cas (PA-GFP-Cas) and photoactivatable mCherry-actin (PA-mCherry-actin). Consistent with our previous observation (Machiyama et al., 2014), the movements of Cas molecules and actin molecules appeared to be correlated in the control CasKO MEFs expressing Cerulean alone (Fig. 3A,B,E; Movie 1). By contrast, the inward movements of Cas molecules were impeded whereas the actin retrograde flow appeared unaffected in CasKO MEFs expressing Cerulean-tensin 1-SH2PTB (Fig. 3C,D,E; Movie 2). These findings indicate that the expression of tensin 1-SH2PTB disrupts the correlation between the actin retrograde flow and the movement of Cas molecules at focal adhesions.

### Exogenous expression of tensin 1-SH2PTB hampers the dissociation of Cas molecules from focal adhesions

**Fig. 4.**
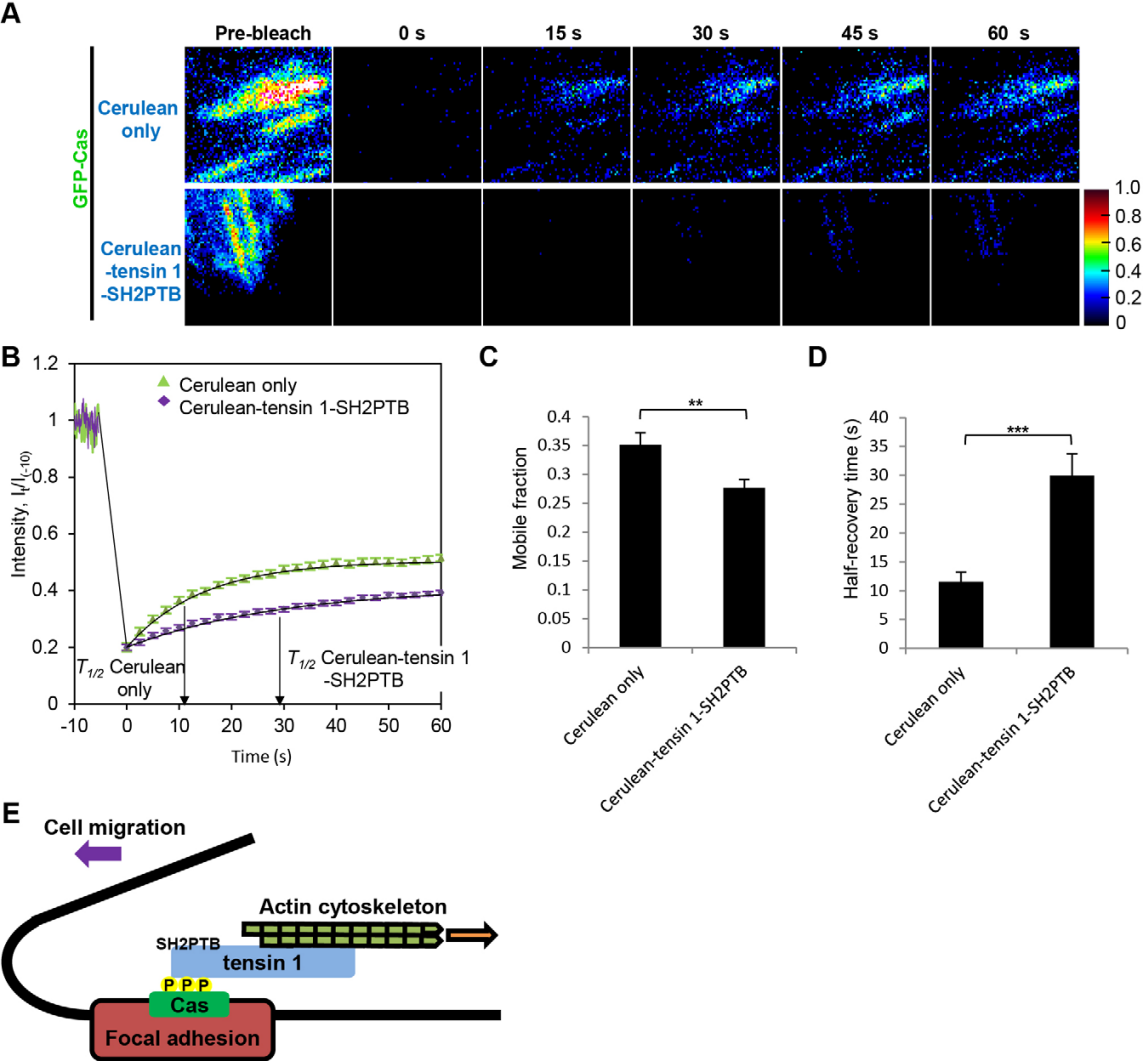
**Exogenous expression of tensin 1-SH2PTB stabilizes Cas residence at focal adhesions.** (A) Representative FRAPs of GFP-tagged Cas expressed in migrating CasKO MEFs co-expressing Cerulean only (top panels) or Cerulean-tensin 1-SH2PTB (bottom panels). Pseudocolor images representing fluorescence intensities of GFP-Cas before (−10 s), immediately (0 s), 15 s, 30 s, 40 s, and 60 s after photobleaching are shown. Scale bar: 1 μm. (B) Mean fluorescence intensities *I(t)/I*_−10_ of individual focal adhesions of cells co-expressing Cerulean only (green triangle) or Cerulean-tensin 1-SH2PTB (purple diamond) are plotted against time. (C,D) Mobile fraction, *M* (C) and half-recovery time, *T_1/2_* (D) were obtained as described in Materials and Methods. Data show the mean±s.e.m.; ***P<0.001; **P<0.01 (*n*>50 in each group); Student's *t*-test. Similar results were obtained from three independent experiments. (E) A model for the actin-Cas linkage. Tensin 1 links phosphorylated Cas to inwardly moving actin cytoskeletons at focal adhesions, thereby supporting cell migration.

To further examine the effect of the exogenous expression of tensin 1-SH2PTB on the dynamic behavior of Cas molecules at focal adhesions of migrating cells from a different aspect, we analyzed the molecular turnover. To this end, we conducted fluorescence recovery after photobleaching (FRAP) experiments using a total internal fluorescence reflection (TIRF) microscope (Machiyama et al., 2014). We found that the recovery of fluorescence from GFP-Cas was significantly encumbered by the exogenous expression of tensin 1-SH2PTB (Fig. 4A,B). When the mobile fraction (*M*) and the half-recovery time (*T_1/2_*) were calculated from the fluorescence recovery curves (Fig. 4B), there was a significant increase in *T_1/2_* as well as a significant decrease in *M* in tensin 1-SH2PTB-expressing cells as compared with their control cells (Fig. 4C,D). These results suggest that the expression of tensin 1-SH2PTB impeded the exchange of Cas molecules at focal adhesions. In particular, the decrease in *M* value relates to the hampered displacement of Cas molecules from focal adhesions (Machiyama et al., 2014) in tensin 1-SH2PTB-expressing cells. By contrast, the turnover rate of paxillin at focal adhesions was not apparently affected by the expression of tensin 1-SH2PTB (Fig. S4). This indicates that the retarding effect of tensin 1-SH2PTB expression on the molecular turnover at focal adhesions is specific for Cas. These results strengthen the notion that tensin 1 links phosphorylated Cas to actin cytoskeletons, and mediates the dissociation of Cas molecules from focal adhesions.

### Src-Cas-tensin 1-actin linkage as a transmission that reconciles the inwardly moving actin filaments and the stationary adhesion complex

In our present study, we identify tensin 1 as the molecule that supports the displacement of Cas from adhesion complexes by linking inwardly moving actin cytoskeletons to phosphorylated Cas (Fig. 4E). Whilst the effect of actomyosin on cellular motility may differ depending on cell types (Kuo et al., 2011) and there appears to be a variation of molecular mechanisms of cell migration, we have previously reported that Cas supports cell migration through the association with Src and actomyosin contraction at focal adhesions (Machiyama et al., 2014). Src tethers Cas molecules to the adhesion complexes and phosphorylates the tyrosine residues in their substrate domains. Phosphorylated Cas then binds to tensin 1 and constitutes a cytoskeleton-adhesion linkage. Thus, continuous actin inward flux leads to the displacement of Cas molecules from the adhesion sites. Based on the temporal nature of Src-Cas and Cas-tensin 1 associations, the Src-Cas-tensin 1-actin linkage could serve as a transmission that reconciles the motile and the stationary parts of the cell, and underpins the locomotive machinery to support the cell migration (Fig. 4E; Movie 3).

## MATERIALS AND METHODS

### Cell culture and retroviral infection

CasKO MEFs and HEK293T cells were cultured in Dulbecco's modified Eagle's Medium (DMEM) (Nissui Pharmaceutical, Tokyo, Japan) containing 10% fetal bovine serum (FBS) (Gibco, Grand Island, NY) supplemented with 100 units/ml of penicillin, 100 mg/ml streptomycin (Gibco), 2 mM L-glutamine (Gibco), and 0.18% sodium bicarbonate under standard cell culture environment at 37°C supplied with 5% CO_2_. Retroviral infection was performed as described previously (Kawauchi et al., 2012).

### Antibodies, chemical reagents and plasmids

Anti-phospho-Cas polyclonal (pCas-165) (Cell Signaling Technology, Danvers, MA), anti-Cas polyclonal (αCas3) (Upstate Biotechnology, Lake Placid, NY), anti-β-actin monoclonal (Sigma-Aldrich, St. Louis, MO), anti-FLAG monoclonal (Sigma-Aldrich), anti-glyceraldehyde-3-phosphate dehydrogenase (GAPDH) (Santa Cruz Biotechnology, Santa Cruz, CA), anti-GFP monoclonal (Clontech Laboratories, Mountain View, CA) antibodies were used for immunoblot, co-immunoprecipitation (Co-IP) and immunofluorescence analyses. Cerulean is a GFP analog and recognized by the anti-GFP antibody, which we used to detect Cerulean-tagged proteins by immunoblotting. All chemicals were purchased from Sigma-Aldrich unless otherwise stated.

The retroviral expression vectors for GFP-CasWT, GFP-Cas15YF and PA-GFP-Cas were constructed as described previously (Machiyama et al., 2014). To generate the expression vectors for Cerulean-tensin 1 variants, chicken tensin 1 was used as a polymerase chain reaction (PCR) template and cloned into pCAGGS (Niwa et al., 1991). The expression plasmids for FLAG-CasWT and FLAG-Cas15YF were constructed using pcDNA3 (Invitrogen) as a parent vector. To prepare the retroviral expression vectors for paxillin-mCherry, PA-mCherry-actin and mCherry-Cas, pBabe-blasticidin was used as a parent vector. To construct the silencing vector for tensin 1, mouse tensin 1 target sequence (5′-GCCTTTGTTTCTGCATCATGT-3′) was cloned into pSuper-puromycin (Oligoengine, Seattle, WA). pET-41 (Novagen, Madison, WI) was used as a parent vector for bacterial expression of GST proteins (GST only and GST-tensin 1-SH2PTB).

### Immunofluorescence analysis

The immunofluorescence staining was performed as described previously (Hirata et al., 2008). The samples were viewed with a confocal microscope (A1R, Nikon) equipped with oil-immersion (NA 1.40, 100×; Plan Apochromat VC, Nikon) and air (NA 0.95, 40×; Plan Apochromat, Nikon) objectives. Acquired images were analyzed off-line with the public domain ImageJ program (NIH).

### Co-immunoprecipitation (Co-IP) assay

Co-IP assay was conducted to biochemically analyze the interaction between FLAG-tagged Cas variants and Cerulean-tagged tensin 1 variants using an anti-FLAG (M2) Affinity Gel (Sigma-Aldrich) according to manufacturer's instructions.

### Adenoviral transduction

The recombinant adenovirus expressing GFP-tensin 1-SH2PTB was generated using the ViraPower Adenoviral Expression System (Invitrogen, Thermo Fisher Scientific, Inc.). GFP only recombinant adenovirus was prepared as described previously (Araki et al., 2013). The adenoviruses were purified with AdEasy Virus Purification Kits (Agilent Technologies, Palo Alto, CA) before use. The purified GFP-tensin 1-SH2PTB adenovirus was then used to transduce the target cell lines at a multiplicity of infection (MOI) of 50 plaque-forming unit (pfu) for 4 days.

### Photoactivation total internal reflection fluorescent speckle microscopy (PA-TIR-FSM)

CasKO MEFs stably co-expressing photoactivatable (PA)-GFP-Cas and PA-mCherry-actin were transfected with Cerulean-tensin 1-SH2PTB or Cerulean only expressing vectors. To record the retrograde flow of actin and the inward movement of Cas simultaneously, PA-TIR-FSM was performed using a Ti-E inverted microscope (Nikon) equipped with an electron multiplying charge coupled device camera with a 512×512-pixel chip (iXon3 897, Andor Technology, Belfast, UK), 1006 NA 1.49 objective lens and fiber-coupled 488-nm and 559-nm lasers to excite GFP and mCherry, respectively. Photoactivation was done by directing laser with 405 nm wavelength onto the cell. The TIRF images of GFP and mCherry were then recorded every 10 s for 25-30 min. NIS elements software (Nikon) was used for image acquisition, and ImageJ software was used for image processing and final figure preparation.

### Wound healing assay

Wound healing assay was performed as described previously (Machiyama et al., 2014). Cells were grown overnight in DMEM containing 10% FBS. A Chamlide magnetic chamber (Live Cell Instrument, Seoul, Korea) with a collagen (10 μg/ml)-coated glass bottom was used to form a monolayer, which was scratched by a pipette tip and viewed with a microscope. Differential interference contrast (DIC) images were taken every 10 min for 10 h. Analysis of migration speed was performed using ImageJ software with a manual tracking plug-in. The displacement of the nuclei at each time point was plotted and the migration rate was quantified by the slope of the plotted points.

### TIRF-FRAP experiments

TIRF-based FRAP (TIRF-FRAP) analysis was conducted as described previously (Machiyama et al., 2014).

### GST pull-down assay and quantitative PCR analysis

GST pull-down assay was performed as described elsewhere (Qian et al., 2009). To measure mRNA expression levels, quantitative PCR analysis was performed as described previously (Kawauchi et al., 2012). The primers used for mouse tensin 1 were 5′-GCCCTCGGCTGTGTATTTAT-3′ (forward) and 5′-AGCCATACAATGGATCGTGA-3′ (reverse). The primers used for mouse GAPDH were 5′-CAACTACATGGTCTACATGTTC-3′ (forward) and 5′-CGCCAGTAGACTCCACGAC-3′ (reverse).
